# Contribution of mRNA Splicing to Mismatch Repair Gene Sequence Variant Interpretation

**DOI:** 10.3389/fgene.2020.00798

**Published:** 2020-07-27

**Authors:** Bryony A. Thompson, Rhiannon Walters, Michael T. Parsons, Troy Dumenil, Mark Drost, Yvonne Tiersma, Noralane M. Lindor, Sean V. Tavtigian, Niels de Wind, Amanda B. Spurdle, the InSiGHT Variant Interpretation Committee, Fahd Al-Mulla

**Affiliations:** Department of Genetics and Bioinformatics, Dasman Diabetes Institute, Kuwait City, Kuwait; Centre for Epidemiology and Biostatistics, Melbourne School of Population and Global Health, The University of Melbourne, Melbourne, VIC, Australia, and Colorectal Oncogenomics Group, Genetic Epidemiology Laboratory, Department of Pathology, The University of Melbourne, Melbourne, VIC, Australia; Institute of Genetics and Molecular Medicine, The University of Edinburgh, Edinburgh, United Kingdom; Institute of Medical Genetics, University Hospital of Wales, Cardiff, United Kingdom; Fondazione Policlinico Universitario A. Gemelli IRCCS, UOC Genetica Medica, Rome, Italy, and Istituto di Medicina Genomica, Università Cattolica del Sacro Cuore, Rome, Italy; Medizinische Klinik und Poliklinik IV, Campus Innenstadt, Klinikum der Universität München, Munich, Germany, and Center of Medical Genetics, Munich, Germany; Woolcock Institute of Medical Research, Sydney, NSW, Australia, and University of Technology Sydney, Sydney, NSW, Australia; Medizinisch Genetisches Zentrum, Munich, Germany; INSERM-U1245, UNIROUEN, Normandy Centre for Genomic and Personalized Medicine, Normandie University, Rouen, France; Genetic Medicine, The Royal Melbourne Hospital, Melbourne, VIC, Australia, and Department of Medicine, The University of Melbourne, Melbourne, VIC, Australia; Department of Tumor Biology, The Norwegian Radium Hospital, Part of Oslo University Hospital, Oslo, Norway; Medizinische Klinik und Poliklinik IV, Campus Innenstadt, Klinikum der Universität München, Munich, Germany, and MGZ - Medical Genetics Center, Munich, Germany; Haukeland universitetssjukehus, Bergen, Norway; The Royal Melbourne Hospital, Melbourne, VIC, Australia; Center for Healthy Aging, Department of Cellular and Molecular Medicine, University of Copenhagen, Copenhagen, Denmark; Department of Clinical Genetics, Leiden University Medical Centre, Netherlands; IngridWinship, Genetic Medicine, The Royal Melbourne Hospital, Melbourne, VIC, Australia, and Department of Medicine, The University of Melbourne, Melbourne, VIC, Australia; ^1^Department of Pathology, The Royal Melbourne Hospital, Melbourne, VIC, Australia; ^2^Department of Clinical Pathology, The University of Melbourne, Melbourne, VIC, Australia; ^3^Genetics and Computational Biology Department, QIMR Berghofer Medical Research Institute, Brisbane, QLD, Australia; ^4^Department of Human Genetics, Leiden University Medical Center, Leiden, Netherlands; ^5^Department of Health Sciences Research, Mayo Clinic, Scottsdale, AZ, United States; ^6^Department of Oncological Sciences, University of Utah School of Medicine, Salt Lake City, UT, United States

**Keywords:** mismatch repair genes, splicing aberrations, variant interpretation and classification, variant type, Lynch syndrome, mRNA splicing

## Abstract

Functional assays that assess mRNA splicing can be used in interpretation of the clinical significance of sequence variants, including the Lynch syndrome-associated mismatch repair (MMR) genes. The purpose of this study was to investigate the contribution of splicing assay data to the classification of MMR gene sequence variants. We assayed mRNA splicing for 24 sequence variants in *MLH1*, *MSH2*, and *MSH6*, including 12 missense variants that were also assessed using a cell-free *in vitro* MMR activity (CIMRA) assay. Multifactorial likelihood analysis was conducted for each variant, combining CIMRA outputs and clinical data where available. We collated these results with existing public data to provide a dataset of splicing assay results for a total of 671 MMR gene sequence variants (328 missense/in-frame indel), and published and unpublished repair activity measurements for 154 of these variants. There were 241 variants for which a splicing aberration was detected: 92 complete impact, 33 incomplete impact, and 116 where it was not possible to determine complete versus incomplete splicing impact. Splicing results mostly aided in the interpretation of intronic (72%) and silent (92%) variants and were the least useful for missense substitutions/in-frame indels (10%). MMR protein functional activity assays were more useful in the analysis of these exonic variants but by design they were not able to detect clinically important splicing aberrations identified by parallel mRNA assays. The development of high throughput assays that can quantitatively assess impact on mRNA transcript expression and protein function in parallel will streamline classification of MMR gene sequence variants.

## Introduction

Loss of function sequence variants in the mismatch repair (MMR) genes causes the cancer susceptibility syndrome, Lynch syndrome. However, for many sequence variants identified, the clinical significance can only be established after considering further evidence, such as population allele frequencies, tumor pathology, family co-segregation information, *in silico* predictions, and experimental assays of MMR function (Thompson et al., 2013a, b, 2014). Some variants are “spliceogenic” and confer pathogenicity by an effect on mRNA splicing, either through the disruption of the native splice sites (5′-donor GT and 3′-acceptor AG), gain of *de novo* sites, activation of cryptic splice sites, or altering splicing regulatory elements (e.g., exonic splicing enhancers and silencers, ESEs and ESSs, respectively) (Cartegni et al., 2002). *In vitro* splicing assays using patient RNA or minigenes are thus often used to test if sequence variants cause splicing defects (Thompson et al., 2015). Output of mRNA splicing assays is incorporated into the MMR gene sequence variant classification scheme developed by the International Society for Gastrointestinal Hereditary Tumours (InSiGHT) Variant Interpretation Committee (Thompson et al., 2014), and the American College of Medical Genetics and Genomics and the Association for Molecular Pathology (ACMG/AMP) guidelines with minimal specifications (Richards et al., 2015). An important consideration in the InSiGHT classification criteria is that allele-specific assays are required to determine the contribution of the variant allele to the overall transcript profile.

Using mRNA splicing assay results from 24 MMR gene variants, and additional splicing data submitted to the InSiGHT and Universal Mutation Databases (UMD), we investigated the utility of splicing assays in the final interpretation of MMR gene variants, considering variant location and predicted effect. We additionally considered the utility of protein functional assay data, where such information was available, for the classification of predicted missense variants.

## Methods

Nucleotide numbering reflects cDNA numbering with +1 corresponding to the A of the ATG translation initiation codon in the reference sequence, with the initiation codon as codon 1. The following GenBank reference sequences were used: *MLH1* – NM_00249.3, *MSH2* – NM_00251.2, *MSH6* – NM_00179.2, and *PMS2* – NM_00535.6.

### Sources of MMR Gene Variants

Cases with MMR gene germline variants (24 unique variants, Supplementary Table S1) in this study were identified from the Colon Cancer Family Registry (CCFR) and the Australian National Endometrial Cancer Study (ANECS) from participants with lymphoblastoid cell lines (LCLs) available for RNA analyses. Both resources have been described previously (Buchanan et al., 2014; Jenkins et al., 2018). Informed consent was obtained from all study participants. All variants interrogated in this study have been submitted to the InSiGHT MMR gene locus-specific databases^1^. Additional clinical data were collected from international sites (through the InSiGHT Variant Interpretation Committee) to aid in variant classification.

### mRNA Analysis

Culturing of CCFR/ANECS case-derived (*n* = 24) and healthy Red Cross donor control-derived (*n* = 12) LCLs in the presence/absence of the nonsense-mediated decay inhibitor puromycin, and RNA extraction and cDNA synthesis were performed as previously described (Whiley et al., 2014). PCR amplification of cDNA from both cases and healthy controls was performed using Mango Taq (Bioline, Eveleigh, NSW, Australia) under the following conditions: 95°C for 2 min followed by 40 cycles of 94°C for 20 s, 60°C for 30 s and 72°C for 1 min and a final extension step at 72°C for 5 min (primer details in Supplementary Table S2). PCR products were separated by agarose gel electrophoresis. Three controls were run alongside each case. Cases and controls showing only single transcripts on gel visualization were sequenced at the Australian Genome Research Facility (Brisbane, QLD, Australia). For products that contained multiple transcripts, the individual bands were excised from the gel and purified using the NucleoSpin Gel and PCR clean up kit (Macherey-Nagel, Düren, Germany) per manufacturer’s instructions. These purified transcripts were then re-amplified before Sanger sequencing. Sequencing chromatograms were visualized using FinchTV (Geospiza, Seattle, WA, United States). The 24 MMR gene variants were also analyzed using multiple *in silico* splicing tools (outlined in Supplementary Table S1).

### CIMRA Assays

A subset of predicted missense substitutions were analyzed for this study using the cell-free *in vitro* mismatch repair activity (CIMRA) assay using techniques previously described for MLH1, MSH2 (Drost et al., 2018), and MSH6 (Drost et al., 2020).

### Dataset Used to Assess Utility of Splicing Assay Data for Classification

All records as of July 2019 that have reported splicing analysis using RNA or minigene assays were extracted from the InSiGHT variant classification database (see text footnote 1), UMD-*MLH1*/*MSH2*/*MSH6* databases (*n* = 162) (Grandval et al., 2013), and various recent publications from which results have since been submitted to the InSiGHT database (Supplementary Table S3). If available, the missense/in-frame indel variants in this set were further annotated with previously generated CIMRA assay data. The five class InSiGHT MMR gene classification scheme was applied if new data were available for previously classified variants and to interpret new variants (Thompson et al., 2014). This incorporated both quantitative (multifactorial likelihood) and qualitative approaches. Multifactorial likelihood analysis was conducted as described previously (Thompson et al., 2013a, b), including the application of recently updated tumor characteristics likelihood ratios (LRs) (Li et al., 2020), and functional LRs. The functional LRs were based on the MMR activity outputs of the MLH1, MSH2 (Drost et al., 2018), and MSH6 (Drost et al., 2020) missense variants from CIMRA assays, represented as percent of wild-type activity. For the purposes of comparing splicing assay and MMR activity assays for missense/in-frame indels, the CIMRA assay data were categorized into deficient, moderate, or proficient function. The thresholds set for deficient and proficient function were equivalent to the probability of pathogenicity cut-offs used for Class 4, likely pathogenic (0.95) and Class 2, likely benign (0.05) derived using the CIMRA assay functional LRs (Thompson et al., 2014; Drost et al., 2018, 2020). The deficient wild-type activity thresholds were set at <23% for MLH1 and MSH2, and <18% for MSH6 and PMS2 (*in lieu* of a calibrated PMS2 functional LR). The proficient wild-type activity thresholds were set at ≥70% for MLH1 and MSH2, and ≥100% for MSH6 and PMS2 (as *PMS2* penetrance is closer to *MSH6* than *MLH1*/*MSH2* (Dominguez-Valentin et al., 2020). If no validated CIMRA data was available for a variant, then the highest published MMR activity assay data value (most conservative) from alternative published assay data extracted from the InSiGHT variant classification database was used as qualitative data points to assign an effect on function. To compare splicing predictions to mRNA results, all sequence variants were annotated with MES-SWA and categorized into groups based on predicted potential to alter splicing, according to guidelines in v2.5 of the ENIGMA consortium *BRCA1/2* variant interpretation criteria^2^ and shown to have 98.7% sensitivity and 96.5% specificity to detect the correct impact on splicing (Shamsani et al., 2018). The groups were as follows, where diff is the difference between the reference and alternate scores and alt refers to the alternate score: *native loss minimal* is diff < 0, or alt > 8.5, or diff < 1.15 and 6.2 ≤ alt ≤ 8.5; *native loss moderate* is diff ≥ 1.15 and 6.2 ≤ alt ≤ 8.5, or diff < 1.15 and alt < 6.2; *native loss high* is diff ≥ 1.15 and alt < 6.2; *gain minimal* is diff > 0, or alt < 6.2, or diff < 0 and 6.2 ≤ alt ≤ 8.5 alt < closest upstream/downstream native splice site; *gain moderate* is diff < 0 and 6.2 ≤ alt ≤ 8.5 alt > closest upstream/downstream native splice site*; gain high* is diff < 0 and alt > 8.5.

### Terminology to Describe Impact of Variants on mRNA Splicing

Variants were placed into one of three categories, determined through Sanger sequencing of cDNA if exonic variant present (method used variants tested in this study) or from other allele-specific techniques:

• Complete impact – variant allele results in expression of only alternatively spliced transcript(s), i.e., no or minimal reference (full-length) transcript is derived from the variant allele,
• Incomplete impact – variant allele results in expression of both reference (full-length) and alternatively spliced transcript(s)
• Extent of impact unknown – a splicing aberration was detected but it was not possible to determine if variant impact was complete or incomplete

## Results and Discussion

**FIGURE 1:**
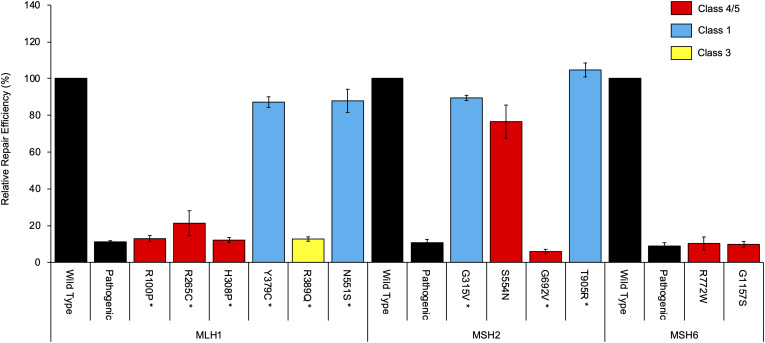
Cell-free *in vitro* mismatch repair activity (CIMRA) assay results for 12 missense substitutions. The MLH1 p.G67R, MSH2 p.A636P, and MSH6 p.G1139S variants are included in every experiment as repair-deficient (pathogenic) controls. Bars represent mean ± SEM of 3–4 experiments. Asterisks indicate substitutions where the CIMRA assay results converted to a functional LR contributed to the final classification of the variant. The color code (red, blue, yellow) refers to the classification of the variants as determined in this study.

mRNA assays were conducted in this study for 24 MMR gene sequence variants. Results are summarized in Table 1 and detailed in Supplementary Table S1 (sequence traces are shown in the Supplementary Figure S1). Results from the CIMRA assay for the 12 presumed missense substitutions are shown in Figure 1.

**TABLE 1:** Summary of splicing assay results from this study and their contribution to variant classification.

Variant	Splicing/transcript expression	Variant splicing impact	InSiGHT class^a^	Splicing contributes to classification?
Missense (*n* = 12)				
*MLH1*				
c.299G > C p.(Arg100Pro)	No effect		5	No
c.793C > T p.(Arg265Cys)	r.791_884del	Complete	5	Yes
c.923A > C p.(His308Pro)	r.[791_1038del, 923a > c]	Incomplete	5^b^	No
c.1136A > G p.(Tyr379Cys)	No effect		1^b^	No
c.1166G > A p.(Arg389Gln)	r.[1039_1409del, 1166g > a]	Incomplete	3	No
c.1652A > G p.(Asn551Ser)	No effect		1^b^	No
*MSH2*				
c.944G > T p.(Gly315Val)	No effect		1^b^	No
c.1661G > A p.(Ser554Asn)	r.1511_1661del	Complete	5^b,c^	Yes
c.2075G > T p.(Gly692Val)	No effect		5	No
c.2714C > G p.(Thr905Arg)	No effect		1	No
*MSH6*				
c.2314C > T p.(Arg772Trp)	r.[628_3172del, 2314c > u]	Incomplete	5	No
c.3469G > A p.(Gly1157Ser)	No effect		4	No
Silent (*n* = 5)				
*MLH1*				
c.438A > G	No effect		2^b^	Yes
*MSH2*				
c.1275A > G	r.[1229_1276del, 1275a > g]	Incomplete	3	No
c.1344C > T	No effect		2^b^	Yes
c.2154A > G	No effect		2	Yes
*MSH6*				
c.3246G > T	No effect		1	No
Splice site (*n* = 3)				
*MLH1*				
c.117-2A > G	r.117_121del	Unknown	5^b^	No
c.589-2A > C	r.589_677del	Unknown	4^b^	No
c.790 + 2T > A	r.678_790del	Unknown	5	No
Intronic (*n* = 4)				
*MLH1*				
c.454-13A > G	r.454_545del	Unknown	4	Yes
*MSH2*				
c.1276 + 11A > G	No effect		2^b^	Yes
c.1511-9A > G	No effect		2^b^	Yes
c.1661 + 5G > C	r.1511_1661del	Unknown	4	Yes

*^a^Updated InSiGHT classification. The current InSiGHT database classifications are in Supplementary Table S3. ^b^New submissions to the InSiGHT database. ^c^Variant would be classified as Class 2, likely benign based on the in silico prior probability with the CIMRA-based functional likelihood ratio. See Supplementary Table S1 for more detail.*

We then assessed the contribution of splicing assay results to final variant classification for 671 MMR gene sequence variants, including the 24 variants assayed for mRNA aberrations from this study (see Supplementary Table S3: *MLH1: n* = 324, 48%; *MSH2: n* = 225, 34%; *MSH6: n* = 73, 11%; *PMS2: n* = 49, 7%). *MLH1* and *MSH2* had the highest proportion of variants assessed, which may be due to their higher penetrance and the increased likelihood of detection using historic Lynch syndrome gene testing guidelines in the clinical setting (Dominguez-Valentin et al., 2020).

There were 156 variants that had not yet been classified by InSiGHT, and 43 variants where new splicing or CIMRA assay data could lead to reclassification from the existing InSiGHT classification. These variants were classified by applying the InSiGHT criteria and have been submitted to the InSiGHT Variant Interpretation Committee for formal classification. Overall, 92 variants caused a splicing aberration designated as complete, 33 variants had incomplete impact (i.e., the full-length transcript was also present), and for 116 variants, it was not possible to determine if impact was complete or not (see Supplementary Table S3).

Of the variants in the acceptor (last 20 bases of intron) or donor (first 6 bases of intron) splice site region, or the first/last 3 bases of the exon (see splice category in Supplementary Table S3), 168/172 with high predicted native splice site loss showed some sort of splicing aberration (98%, three of these were designated incomplete and one variant was reported as complete and incomplete in two separate studies). Another 12/15 with moderate predicted native splice site loss showed an aberration (80%, impact for one variant was designated complete and incomplete splicing in two separate studies). Splicing impact was seen for 11/52 variants with minimal predicted native loss (21%, three reported as incomplete); 4/11 were exonic variants that led to complete exon skipping events, which may be due to an effect on ESE or ESS that are not predicted by the MES-SWA tool, or otherwise false negative native loss predictions.

For the *de novo* donor/acceptor gain predictions, 13/26 variants with high predicted gain showed effect on mRNA splicing aberration (50%); of these, three had incomplete impact: one was a predicted stop gain variant, and two were confirmed to also have an effect on function due to the predicted missense change. Splicing impact was observed for 3/7 (43%) of variants with moderate predicted gain, one of which demonstrated complete activation of a cryptic splice site (*MSH2* c.2635-1G > T). Of the remaining two variants, one had high predicted native loss (*MLH1* c.1039-2A > T) and the third had no predicted effect on the native splice site (*MSH2* c.1979A > G).

Splicing alterations were reported for 225/638 (35%) of variants with no/minimal predicted gain, with splicing impact due to alternative mechanisms. The vast majority of these (176/225) were located in the splice region (defined as above—last 20 bases of the intron, first 6 bases of the intron, or the first/last 3 bases of the exon) with moderate-high prediction of native site loss, and the remainder were largely exonic variants with incomplete exon skipping events (26/49)—again implying effect on ESE/ESS.

Overall, these findings highlight the complexities of using splice site prediction algorithms to prioritize variants for potential splice assays. Prediction relating to both native site loss and *de novo* gain need to be considered in parallel to assess if a variant is potentially spliceogenic, and to consider variant location in/near a splice site. Nevertheless, it is clear that triage of variants based on location in the splice region provides the most efficient method to detect spliceogenic variants. Our findings also emphasize a known deficiency in variant annotation with respect to potential effect on ESE/ESS, due to the poor specificity of currently available prediction tools (Houdayer et al., 2008). This observation stresses the importance of considering all available points of evidence (clinical and functional) to inform variant interpretation.

**FIGURE 2:**
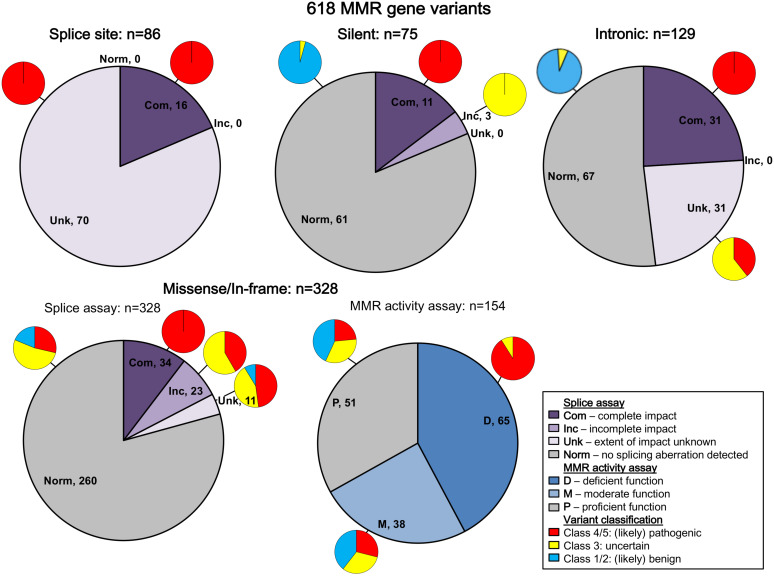
Summary of contribution of splicing assay data to variant classification. The figure legend describes the categorization of the splicing and MMR activity assay data. Splice site refers to variants in the conserved IVS ± 1/2 dinucleotides of the acceptor or donor splice site, while intronic includes all other intronic variants. Predicted loss of function variants other than splice site variants were excluded from this summary because they are classified as pathogenic regardless of splicing assay results (frameshift: *n* = 25; nonsense: *n* = 24). There were also two initiation codon variants and two stoploss variants not included in this summary. Com, complete impact, variant allele causes complete splicing aberration; D, deficient function (MLH1/MSH2: <23% wild-type repair, MSH6/PMS2: <18% wild-type activity); Inc, incomplete impact, variant allele results in expression of both reference (full-length) and alternatively spliced transcript(s); M, moderate function (MLH1/MSH2: 23% to <70% wild-type repair, MSH6/PMS2: 18 to <100% wild-type activity); Norm, no splicing aberration detected; P, proficient function (MLH1/MSH2: ≥70% wild-type repair, MSH6/PMS2: ≥100% wild-type activity); Unk, extent of impact unknown, splicing aberration detected, but unable to determine if variant impact was complete/incomplete.

All variants were assigned to categories based on variant type. The results are summarized in Figure 2 (and described in more detail in Supplementary Table S3). Bearing in mind that *in vitro* experiments were likely prioritized by splicing predictions for individual variants, the results show that splicing assay results informed classification most for silent variants (92%; 69/75) and intronic variants (72%; 93/129), and least for missense substitutions/in-frame indels (10%; 34/328).

All native splice site dinucleotide variants assessed (*n* = 86) caused splicing aberrations. However, levels of the splicing aberration from the variant allele were reported for only 16 variants, information which alone permits upgrade from likely pathogenic class to pathogenic class, in accordance with InSiGHT classification criteria. Due to their very high likelihood to alter splicing, variants altering the canonical intronic dinucleotides at the native splice sites were traditionally considered pathogenic without the need to conduct splicing assays (Thompson et al., 2014; Abou Tayoun et al., 2018), but this mindset is no longer held given that consideration of naturally occurring splicing, and the predicted mRNA product is now recognized as an important aspect of variant curation (de la Hoya et al., 2016; Abou Tayoun et al., 2018). There are currently no exceptions (due to consideration of naturally occurring “rescue” isoforms) that have been identified in the MMR genes.

Splicing information was most likely to contribute evidence against pathogenicity for synonymous/silent and intronic variants, with 61/75 (81%) and 67/129 (52%) demonstrating the absence of a splicing aberration, respectively. This includes five intronic and three silent variants that demonstrated no impact on splicing, but are classified as VUS because NMD inhibitors were not used in the splicing analysis, which is a requirement for the InSiGHT splicing interpretation criteria. For these variant types, effects on splicing (or perhaps overall transcript expression) are the most likely causes of loss of function (Parmley and Hurst, 2007; Parmley and Huynen, 2009).

We did not find splicing data as useful in the interpretation of predicted missense substitutions; 68/328 (21%) of predicted missense/in-frame alterations altered mRNA splicing. Of these 68 proven spliceogenic variants, the mRNA splicing data contributed to the classification of only 34 variants (50%; due to detection of complete splicing that was considered as evidence toward pathogenicity). Further, this observation likely overestimates the proportion of predicted missense variants that (also) alter mRNA splicing; bias toward spliceogenic variants having undergone mRNA assays is anticipated given that bioinformatic prediction of potential effect on splicing is commonly used to prioritize selection of variants for splicing assays in the research and clinical setting. Indeed, 37/68 (54%) of spliceogenic missense variants had high-moderate predicted potential to affect splicing using splicing prediction performed here, which focused on impact on native splice sites, or creation of *de novo* or activation of cryptic splice sites (but excluded prediction of effect on exonic splicing regulators, i.e., ESEs and ESSs). As might be expected, MMR activity assays were more useful to support classification of missense substitutions/in-frame indels as pathogenic, with 59/65 (91%) of variants with deficient MMR activity being classified as Class 4/5 (likely) pathogenic (Figure 2 and Supplementary Table S3). Thus, MMR activity functional assays are more useful in the interpretation of missense/in-frame indels, particularly now the output of CIMRA can be used in quantitative multifactorial analysis (Drost et al., 2018, 2020).

The current MMR activity assays do not detect impact on all biological effects; indeed, there were four (likely) pathogenic *MLH1* variants with proficient MMR activity and normal splicing (p.Lys618del, p.Pro640Ser, p.Ala681Thr, and p.Arg687Trp). For these variants, the probable cause of pathogenicity is a defect not measured by either the CIMRA assay or the splice assays reported here, such as that related to cellular localization, protein instability, or DNA damage-response. Further, current MMR activity assays are cDNA-based and cannot detect aberrant splicing; there were seven pathogenic missense variants with proficient MMR activity, where the nucleotide substitution caused complete expression of a splicing aberration. Of the (likely) benign variants, none had deficient MMR activity, and one had moderate MMR activity.

These observations of “conflicting” mRNA splicing and protein functional assays suggest that alternative approaches, which combine assessment of effects at the mRNA and protein level, are required to simplify interpretation on laboratory assay data for MMR gene variant classification. The assay recently developed for BRCA1 (Findlay et al., 2018), saturation genome editing followed by mRNA expression and cellular loss of function, has demonstrated the feasibility and utility of such combined assays for variant interpretation. However, this specific approach would have to be adapted to account for the fact that unlike BRCA1, the MMR genes are not essential (Blomen et al., 2015). In this regard, an assay based on gene editing of human embryonic stem cells and assessment of both DNA damage response and microsatellite repair was recently developed, holding great promise for the study of variant-induced splicing changes and missense alterations in Lynch syndrome (Rath et al., 2019).

There were 33 variants that demonstrated incomplete impact with respect to expression of aberrant transcripts (see Figure 2 and Supplementary Table S3). Seven of these were frameshift/nonsense variants for which mRNA products are expected to undergo NMD, and thus classification of these variants as pathogenic is unaltered by the mRNA findings. Another 23 were exonic predicted missense/in-frame alterations of the translated protein; protein assay data available for 15/23 variants showed that nine had clear impact on function due to the missense alteration, and another two had moderate function considered to be borderline deficient. That is, protein assay results would inform classification in favor of pathogenicity for 9/15 variants irrespective of the equivocal nature of the mRNA results. Three silent variants (located in the last 3 bp of the exon) and an intronic variant located in the splice donor motif also demonstrated incomplete impact on mRNA splicing, which did not contribute to their classification.

It will be necessary to determine, for variants with incomplete impact on mRNA splicing, what proportion of alternatively spliced MMR gene transcript arising from a variant allele will or will not confer pathogenicity *in vivo*, where a second somatic hit may play a role. It has been shown that a *BRCA1* spliceogenic variant resulting in 70–80% expression of a non-functional transcript (de la Hoya et al., 2016) is not risk-associated. There is some evidence to suggest that the tolerable level of expression may be similar for *MSH2*; *MSH2* c.1275A > G, reported to be associated with 70% expression of aberrant transcript r.[1229_1276del, 1275a > g] (Morak et al., 2019), is currently classified as a VUS but with accumulating clinical evidence trending toward likely benign. While, evidence from a knock-down study assessing correlation between total mRNA expression levels and MMR protein relative repair activity in human fibroblast cell lines (Kansikas et al., 2014) indicates that ∼25% *MLH1* or *MSH2* mRNA expression results in abrogated repair activity. However, it is difficult to interpret the relevance of these apparently conflicting findings in the context of tumorigenesis *in vivo*. We conclude that further research is necessary to elucidate the relationship between MMR gene transcript expression level in human cells and disease risk.

Methods that enable quantification of the proportion of aberrantly spliced transcripts arising from a variant allele, such as recently developed RNA massively parallel sequencing assays (Farber-Katz et al., 2018; Karam et al., 2019), will aid in the interpretation of cases that demonstrate expression of naturally occurring alternatively spliced transcripts and greatly improve the contribution of splicing assays to classification of sequence variants once methods for quantifying transcript expression are routinely instituted. These assays will further increase the use and utility of splicing assay data in variant classification by fulfilling the requirement of quantifying the splicing defect to ensure no full-length transcript is expressed, as currently documented in the InSiGHT MMR gene classification rules (Thompson et al., 2014). This will be particularly useful as supporting clinical data are harder to obtain as more variants of uncertain significance are identified through higher throughput clinical gene panel testing.

**FIGURE 3:**
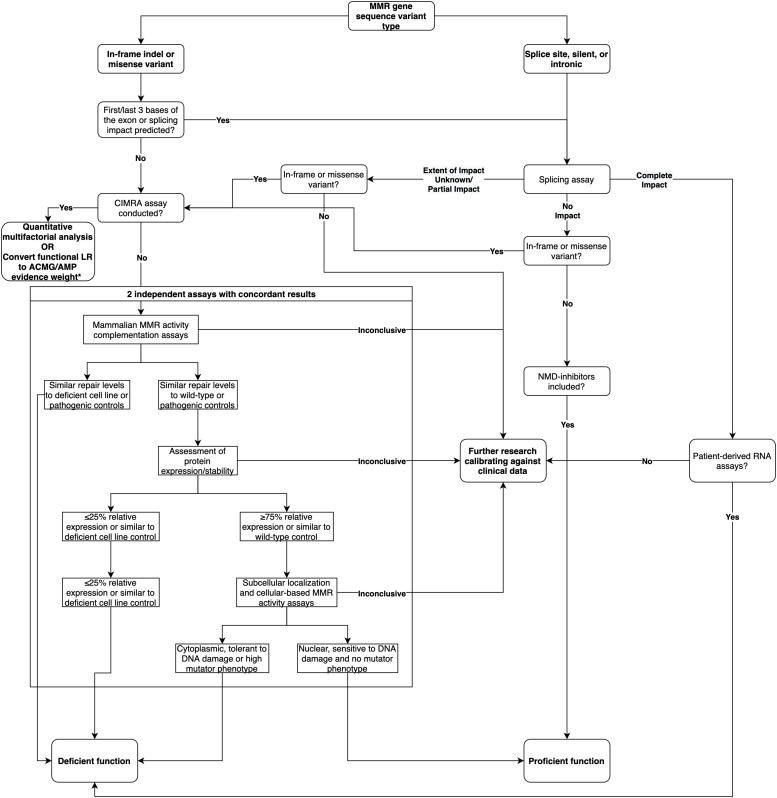
Decision tree for the recommended course of action when assessing the functional impact of MMR gene variants, updated from the decision tree published in Thompson et al. (2014). * As per likelihood ratio or odds for pathogenicity cut-offs reported by Tavtigian et al. (2018) and Brnich et al. (2019).

In summary, based on the analysis of this dataset, we show that splicing assays are a useful adjunct to the interpretation of intronic and silent variants. While mRNA analysis can contribute to the classification of predicted missense/in-frame indel variants, results have to be considered in parallel with data from MMR activity assays. Based on these findings, we provide a decision tree for the recommended course of action when assessing the functional impact of MMR gene variants (Figure 3). We conclude that there is need to develop and validate different high throughput assays that can measure variant effects on cellular function due to mRNA transcripts and/or protein function—due to a variety of biochemical effects—to streamline future MMR gene variant classification.

## Members of the InSiGHT Variant Interpretation Committee

Fahd Al-Mulla, Department of Genetics and Bioinformatics, Dasman Diabetes Institute, Kuwait City, Kuwait; Daniel Buchanan, Centre for Epidemiology and Biostatistics, Melbourne School of Population and Global Health, The University of Melbourne, Melbourne, VIC, Australia, and Colorectal Oncogenomics Group, Genetic Epidemiology Laboratory, Department of Pathology, The University of Melbourne, Melbourne, VIC, Australia; Susan Farrington, Institute of Genetics and Molecular Medicine, The University of Edinburgh, Edinburgh, United Kingdom; Ian Frayling, Institute of Medical Genetics, University Hospital of Wales, Cardiff, United Kingdom; Maurizio Genuardi, Fondazione Policlinico Universitario A. Gemelli IRCCS, UOC Genetica Medica, Rome, Italy, and Istituto di Medicina Genomica, Università Cattolica del Sacro Cuore, Rome, Italy; Elke Holinski-Feder, Medizinische Klinik und Poliklinik IV, Campus Innenstadt, Klinikum der Universität München, Munich, Germany, and Center of Medical Genetics, Munich, Germany; Maija R. J. Kohonen-Corish, Woolcock Institute of Medical Research, Sydney, NSW, Australia, and University of Technology Sydney, Sydney, NSW, Australia; Andreas Laner, Medizinisch Genetisches Zentrum, Munich, Germany; Alexandra Martins, INSERM-U1245, UNIROUEN, Normandy Centre for Genomic and Personalized Medicine, Normandie University, Rouen, France; Finlay Macrae, Genetic Medicine, The Royal Melbourne Hospital, Melbourne, VIC, Australia, and Department of Medicine, The University of Melbourne, Melbourne, VIC, Australia; Pål Møller, Department of Tumor Biology, The Norwegian Radium Hospital, Part of Oslo University Hospital, Oslo, Norway; Monika Morak, Medizinische Klinik und Poliklinik IV, Campus Innenstadt, Klinikum der Universität München, Munich, Germany, and MGZ – Medical Genetics Center, Munich, Germany; Elisabet Ognedal, Haukeland Universitetssjukehus, Bergen, Norway; John-Paul Plazzer, The Royal Melbourne Hospital, Melbourne, VIC, Australia; Lene Juel Rasmussen, Center for Healthy Aging, Department of Cellular and Molecular Medicine, University of Copenhagen, Copenhagen, Denmark; Carli Tops, Department of Clinical Genetics, Leiden University Medical Centre, Netherlands; Ingrid Winship, Genetic Medicine, The Royal Melbourne Hospital, Melbourne, VIC, Australia, and Department of Medicine, The University of Melbourne, Melbourne, VIC, Australia.

## Data Availability Statement

The raw data supporting the conclusions of this article will be made available by the authors, without undue reservation, to any qualified researcher.

## Ethics Statement

The studies involving human participants were reviewed and approved by QIMR Berghofer Human Research Ethics Committee. The patients/participants provided their written informed consent to participate in this study.

## Author Contributions

BT and AS contributed to the conception and design of the study. BT, RW, MP, TD, MD, YT, NL, NW, and ST contributed to the data acquisition and interpretation of the study. BT performed the data analysis and wrote the first draft of the manuscript. BT and AS wrote the sections of the manuscript. All authors contributed to manuscript revision, read, and approved the submitted version.

## Conflict of Interest

ST holds Illumina stock in a personally managed account. The remaining authors declare that the research was conducted in the absence of any commercial or financial relationships that could be construed as a potential conflict of interest. The reviewer CH declared a past co-authorship with one of the author ST to the handling editor.

## Footnotes

1 https://www.insight-database.org

2 https://enigmaconsortium.org/
