# Supplementary material for: Contribution of mRNA Splicing to Mismatch Repair Gene Sequence Variant Interpretation
Source: Front Genet. 2020 Jul 27;11:798. doi: 10.3389/fgene.2020.00798 (PMC7398121; doi:10.3389/fgene.2020.00798)

Supplementary Figure. Sequence traces showing aberrant/alternative splicing

MLH1 c.117-2A>G

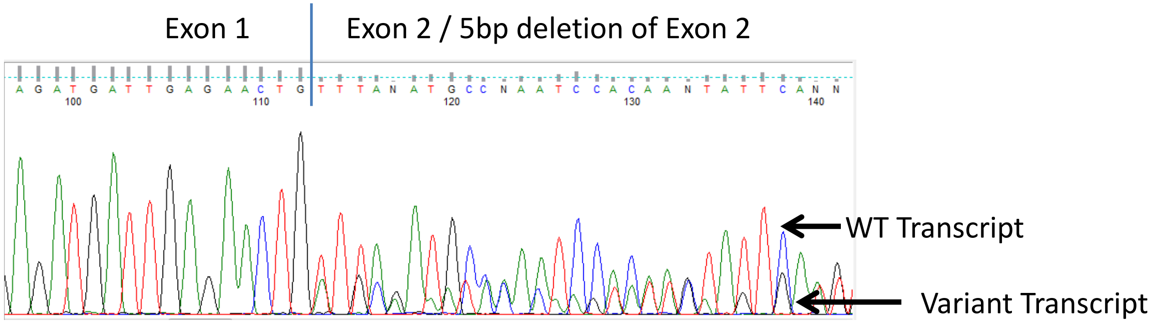

MLH1 c.454-13A>G

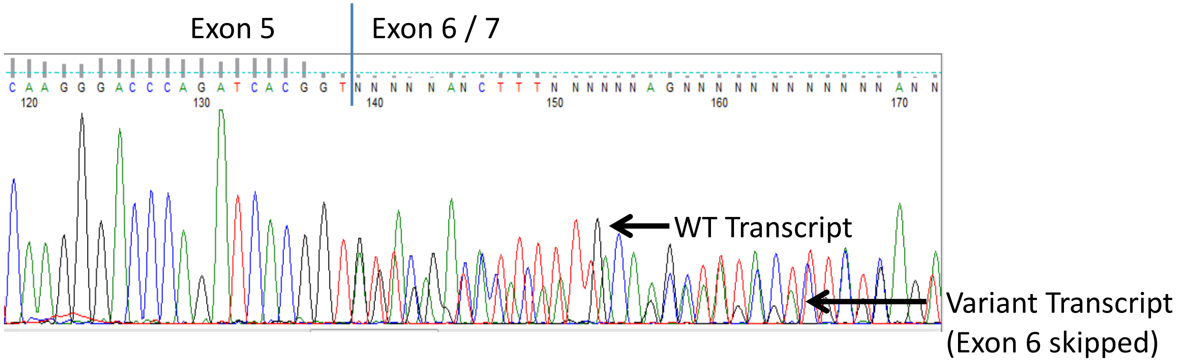

MLH1 c.790+2T>A

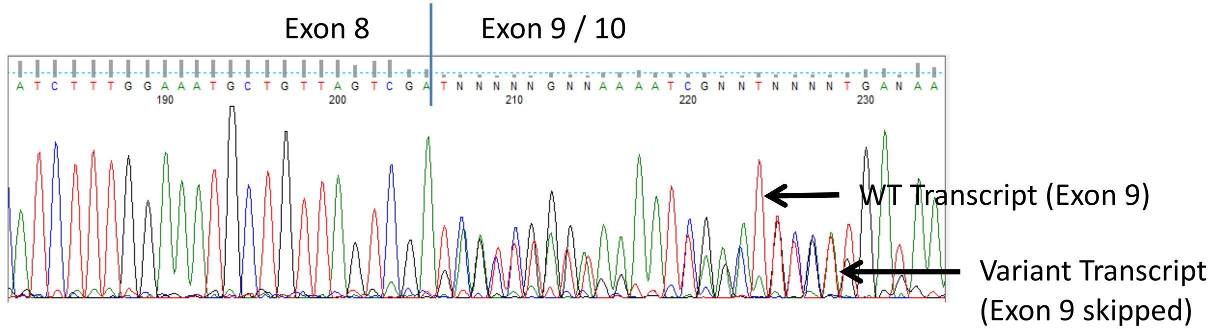

MLH1 c.923A>C

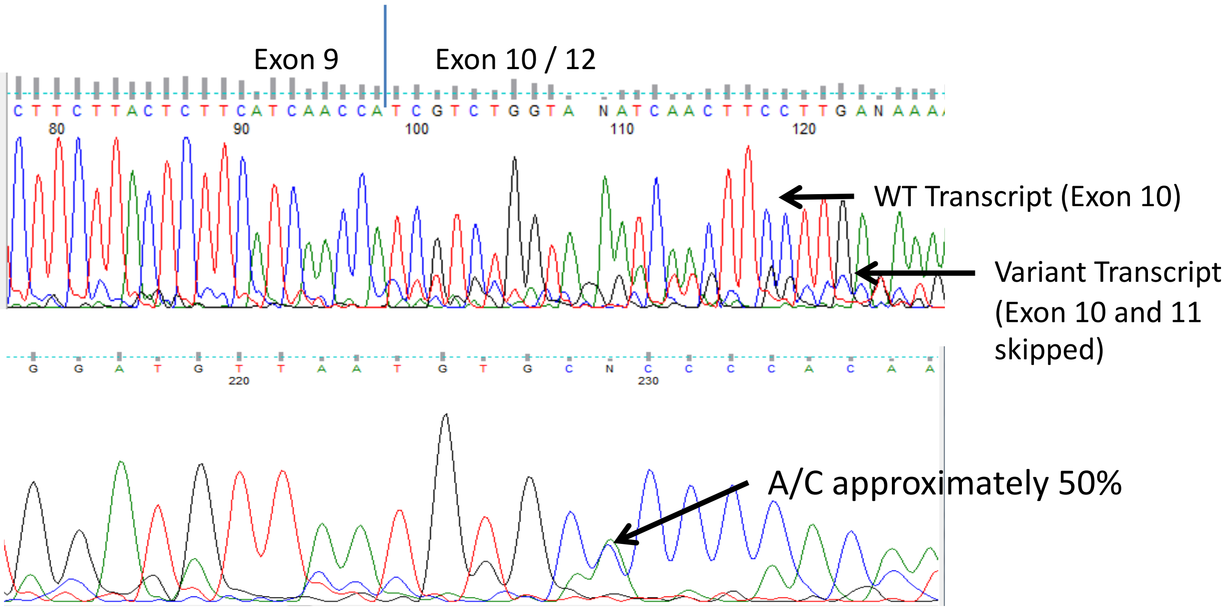

Control

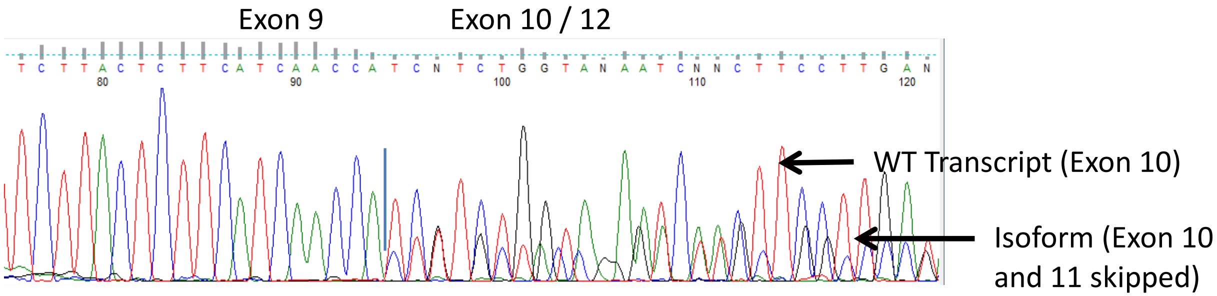

MLH1 c.1166G>A

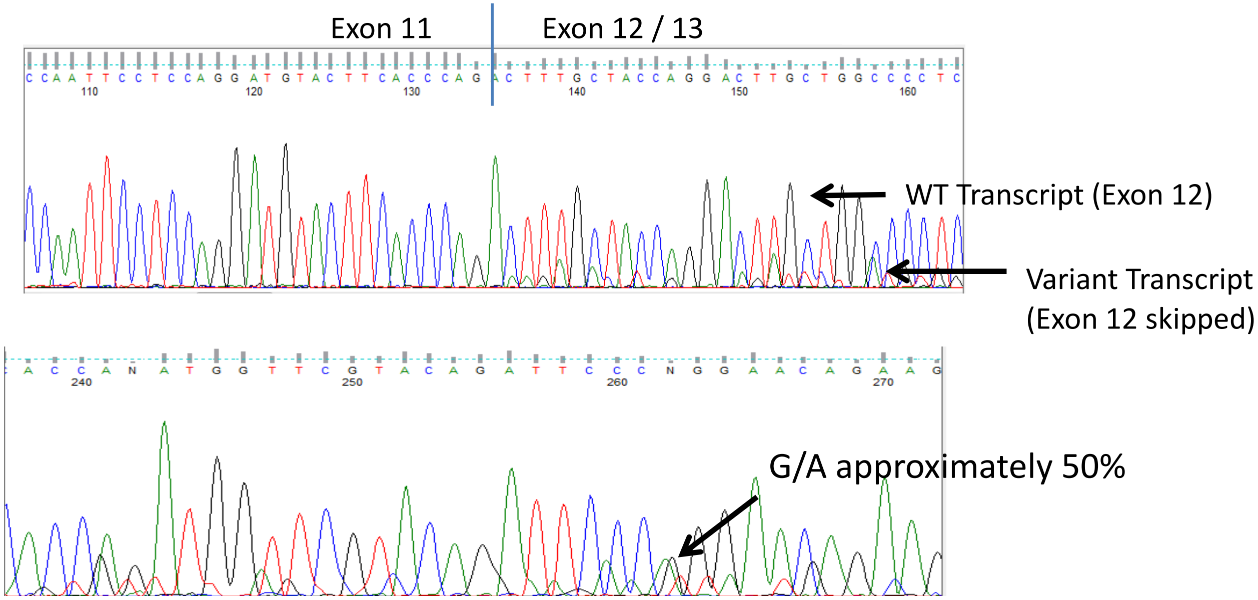

MSH2 c.1275A>G

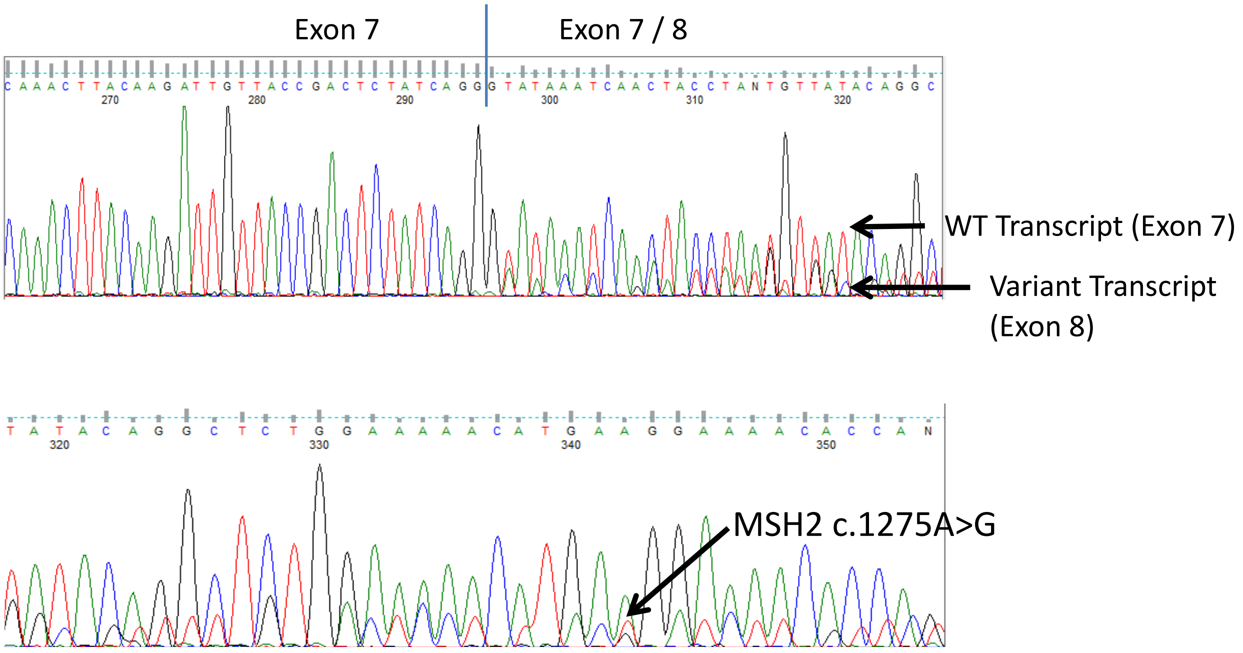

MSH2 c.1661+5G>C

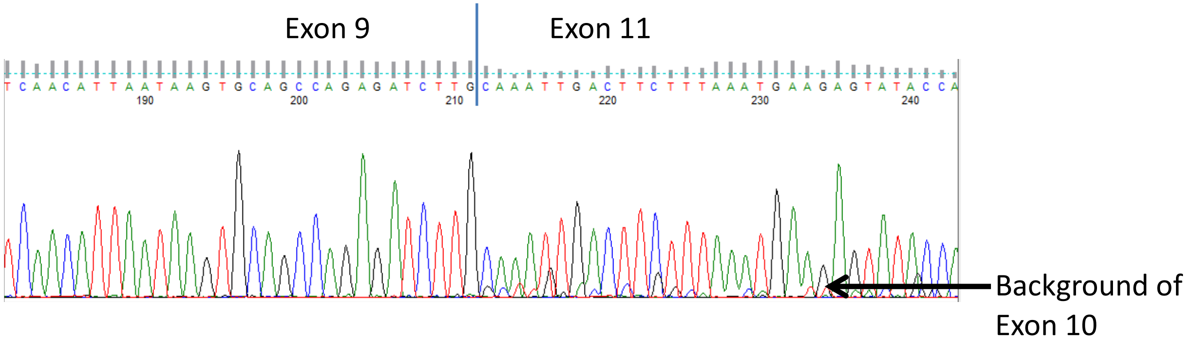

MSH6 c.2314C>T

(Exon 4 skipped in Carrier and Control)

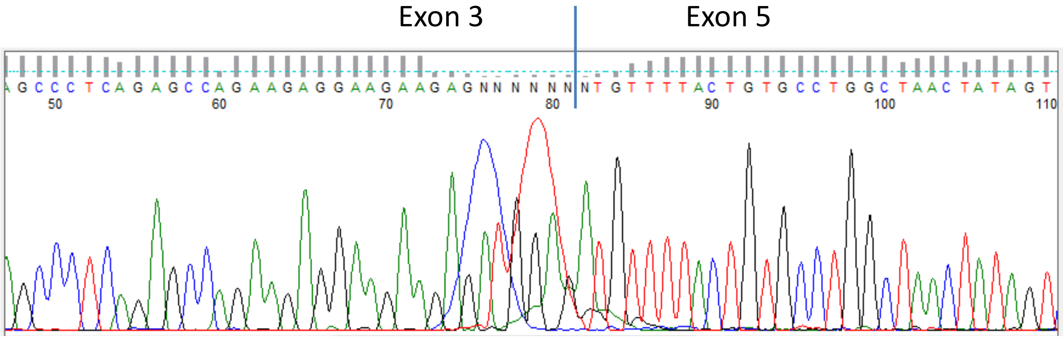

Wild Type Transcript in variant carrier

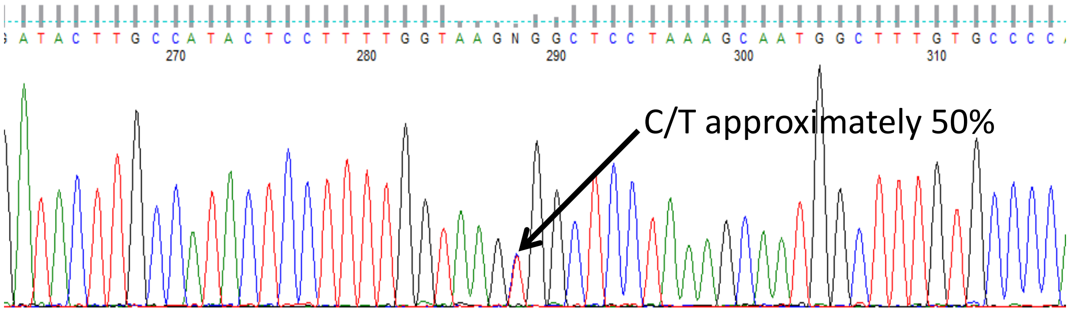

Supplement: Supplementary file 1 [file Image_1.pdf]
